# Intelligent Optimization Method of Resource Recommendation Service of Mobile Library Based on Digital Twin Technology

**DOI:** 10.1155/2022/3582719

**Published:** 2022-08-27

**Authors:** Shanshan Shang, Zikai Yu, Aili Geng, Xiuxiu Xu, Huizhen Ma, Guozhong Wang

**Affiliations:** ^1^Library, Shanghai University of Engineering Science, Shanghai 201620, China; ^2^Assembly Department, Shanghai Aerospace Equipments Manufacturer Co., Ltd., Shanghai 200245, China; ^3^Institute of Artificial Intelligence Industry, Shanghai University of Engineering Science, Shanghai 201620, China

## Abstract

In order to improve the Resource Recommendation and sharing ability of mobile library, an intelligent optimization model of Mobile Library Resource Recommendation Service Based on digital twin technology is proposed. Build the association rule feature distribution set of mobile library resource recommendation service, carry out text information retrieval in the process of Mobile Library Resource Recommendation and sharing, carry out semantic correlation feature registration according to the retrieval preference of mobile library reading user object, establish the association rule data set of mobile library reading user object preference for mobile library Resource Recommendation and sharing, carry out feature block processing, and analyze the library reader preference. Complete the collaborative filtering recommendation of Mobile Library Resource Recommendation sharing. The simulation results show that the collaborative recommendation under the intelligent optimization mode of mobile library resource recommendation service using this method has high accuracy and good confidence level, which improves the intelligent level of Mobile Library Resource Recommendation and user satisfaction.

## 1. Introduction

As a place with strong professional scholarship and comprehensive scientific research, mobile library has provided a huge development space for discipline teaching, cultural exchange, scientific research and application technology in Colleges and universities [1, 2]. In order to enable the library to play a greater social role, improve its social value of information services and the quality of information service products, considering how to improve the sociality of services is also an important opportunity for the quality improvement and career development of library staff [3, 4]. The research on Collaborative Recommendation Algorithm under the intelligent optimization model of mobile library resource recommendation service is of great significance in the construction of digital library. The related research on intelligent optimization model and fusion scheduling algorithm of mobile library resource recommendation service has attracted great attention.

Reference [5] takes the public library in Matara District of Sri Lanka as an example, and puts forward the research on mobile library services of public libraries. The main purpose of this research is to investigate the application, practice, and challenges of mobile library services provided by public libraries. The survey method is used as the data collection method. The library does not collect books separately for the mobile library service, and the mobile library has fewer books. The mobile library identifies school-age children as its target audience, and most people use the vehicles of management institutions to transport books and provide services. Research shows that no library provides this service to people in hospitals and prisons, and the vehicles allocated to mobile libraries lack the necessary facilities. Increasing financial support, the number of books collected, publicity plans, and improving transportation facilities can be used as suggestions. Reference [6] proposes a mobile library Resource Recommendation Algorithm Based on data mining, which uses block fusion clustering analysis and data mining algorithm combined with particle swarm optimization to analyze the personalized demand feature categories of users for mobile library resources, and realizes mobile library resource recommendation according to the identification of typed parameters. This method has poor recommendation performance in complex environments.

In view of the above problems, this paper proposes an intelligent optimization model of Mobile Library Resource Recommendation Service Based on digital twin technology. Combined with the filter detection method, it realizes the analysis of Library Readers' preference, realizes the collaborative matching of Mobile Library Resource Recommendation sharing and reading user object preference, and completes the collaborative filtering recommendation of Mobile Library Resource Recommendation sharing. Finally, simulation experiments are carried out to show the superior performance of this method in improving the collaborative recommendation ability under the intelligent optimization model of mobile library resource recommendation service.

## 2. Theoretical Method of Digital Twinning Technology

Digital twin technology is an integrated system composed of data, models, and analysis tools. It can not only describe the state of aircraft fuselage in the whole life cycle but also make operation and maintenance decisions (including real-time diagnosis and future prediction) for the whole fleet and single fuselage based on uncertain information [7, 8].

**Figure 1:**
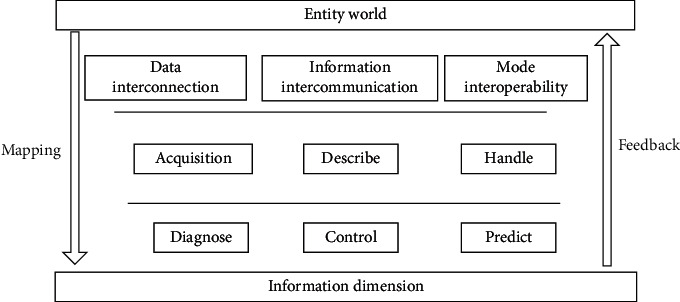
Digital twin functional architecture.

Digital twinning is a virtual entity that creates physical entities digitally. It makes full use of data such as physical model, sensor update and operation history [9, 10], integrates multi-disciplinary, multi-physical, multi-scale and multi-probability simulation processes, and completes mapping in virtual space, thus reflecting the whole life cycle process of the corresponding physical equipment [11, 12]. By digitizing all elements of the physical world, such as people, things, and events, a corresponding “virtual world” will be recreated in cyberspace, forming a pattern of coexistence of physical world and digital world in the information dimension [13, 14]. The dynamics of the physical world are fed back to the digital world accurately and in real time through sensors [15]. Digitization and networking can realize the goal of turning reality into reality, networking and intelligence can realize the goal of turning reality into reality, and through virtual-real interaction and continuous iteration, the best and orderly operation of the physical world can be realized. It is the core element of digital twinning. It originates from physical entities, virtual models, and service systems, and at the same time, it is integrated into each part after integration, which promotes the operation of each part [16–18]. Therefore, data acquisition is the basis of digital twinning. To read the equipment data from the control system, it is necessary to clean the native data through data format analysis, data structure redefinition, data logic redefinition, etc., and then extract the key and effective parts from the data and output them. Twin data include data related to physical entities, virtual models, service systems, domain knowledge, and their fusion data, and is constantly updated and optimized with the production of real-time data. Twin data are the core driver of digital twin operation. The above four parts are connected pairwise to enable effective real-time data transmission, thus realizing real-time interaction to ensure consistency and iterative optimization among the parts. Digital twinning technology supports the open communication standard OPCUA and API custom protocol access, which can ensure the stability of data transmission, reduce the delay of data transmission, realize the high speed, high reliability, and high adaptability of edge data acquisition, and provide an important foundation for subsequent digital twinning applications. The digital function architecture is shown in Figure 1.

It can be seen from Figure 1 that the digital twin functional architecture is a simulation process that makes full use of the physical world, information dimensions and other information to map and feedback each other, integrates multi-disciplinary, multi-physical quantities, multi-scale and multi-probability, and completes the mapping in the virtual space, so as to reflect the whole life cycle process of the corresponding real equipment. Digital twinning is a concept that transcends reality and can be regarded as a digital mapping system of one or more important and interdependent equipment systems.

## 3. Information Retrieval and Information Extraction of Mobile Library Resource Recommendation

### 3.1. Overall Structure and Information Retrieval of Mobile Library Resource Recommendation

In order to realize the optimization design of intelligent optimization model of mobile library resource recommendation service based on digital twin technology, firstly, the information retrieval model of mobile library resource recommendation sharing is constructed, and the personalized preference judgment model of mobile library resource recommendation is established. This paper analyzes the local relationship between users and the library, and then explores the diversity of users' preferences. Hidden factor analysis is a model-based collaborative filtering method, which defines the recommendation problem as the behavior matrix between sparse users and libraries. Assuming the low-rank matrix, the low-rank approximation of the matrix is performed to realize the learning task of implicit representation [19]. The hidden factor analysis method based on matrix approximation can effectively estimate the global structure of users' personalized preference judgment. Split the original behavior matrix into multiple sub-matrices, where each sub-matrix contains the relevance of users, projects, and ratings. The recommended method expression based on matrix approximation is

(1) $$\begin{array}{c} V_{m}=\frac{C_{m}\times \chi }{\rho }. \end{array}$$

In formula (1), *C*_*m*_ represents the set of fitting real score items, *χ* represents the number of users, and *ρ* represents the parameter of loss items. Implicit representation of user preference diversity by low-rank matrix approximation:

(2) $$\begin{array}{c} Q=\sum V_{m}\times g\times \left(1-j\right). \end{array}$$

In formula (2), *g* represents the preference weight of the user, and *j* represents the option of fitting the scoring items. Two-stage separated local low-rank matrix approximation is used to weight the results of user preference diversity:

(3) $$\begin{array}{c} H_{i}=\frac{{\left[Z\left(x_{i}\right)-Z\left(x_{i}+h\right)\right]}^{2}}{Q}. \end{array}$$

**Figure 2:**
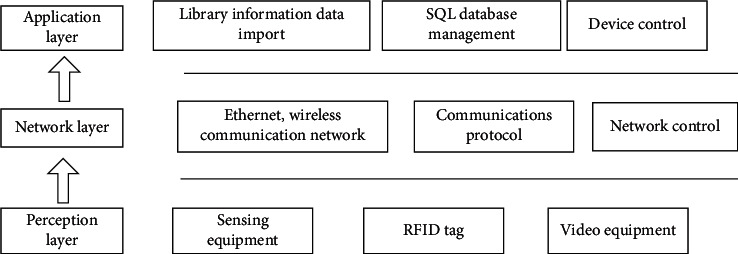
Information retrieval and intelligent service structure system of resource recommendation and sharing in mobile library.

In formula (3), *Z* represents the superposition of nonlinear fitting functions, *x*_*i*_ represents the item score of the *i*-th user, and *h* represents the dependence of missing data. The global low-rank matrix approximation method can effectively obtain the overall characteristics of user preference diversity. According to the correlation analysis of users, items, and ratings, the model factors of personalized distribution of mobile library resources are established, and the tag form of mobile library resource recommendation is obtained: *G* = (*V*, *E*, *C*). Where *V* represents the nearest neighbor set of graph model nodes recommended by mobile library resources, and the RFID tag of each node represents the number of items of mobile library resources; *E* represents the edge set of the distribution domain of mobile library resources, and represents the distribution sequence of user parameters of mobile library resources; Indicates the user's interest in different mobile library resources. Using the method of knowledge map modeling analysis, combined with personalized feature preferences, this paper establishes the user preference typing parameters of mobile library resource recommendation, analyzes the click and collection distribution characteristics of mobile library resource user *U* in a period of time, optimizes the collection management of library resources by combining video monitoring equipment, realizes the integration and information transmission of library resource information in the network layer, and constructs the library information management database in the application layer. SQL database is used to build the local database of library resource information management, artificial intelligence algorithm is used to manage and optimize the scheduling of information, and man-machine interaction is carried out in the application layer to realize the information retrieval and intelligent service of mobile library resource recommendation and sharing [20]. The information retrieval and intelligent service mode of mobile library resource recommendation and sharing designed in this paper is shown in Figure 2.

**Figure 3:**
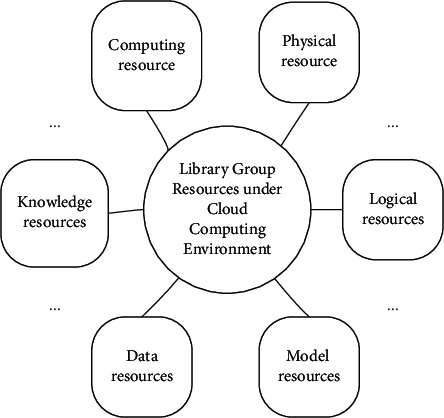
Resource distribution structure model of resource recommendation and sharing in mobile library.

According to the distribution structure model of mobile library resources recommendation and sharing resources shown in Figure 2, the phase space reconstruction method is adopted to reconstruct and extract the features of library resources under the intelligent service mode. Phase space reconstruction is a method to recover and describe the motive power system from the known time series. Using takens' delay embedding theorem, the infinite and noiseless dimension factors are embedded into the phase space. Multi-dimensional phase space vector is constructed by different delay times of one-dimensional time series. Reconstruct a phase space from one-dimensional chaotic time series, which is the same as the power system in topological sense, and judge, analyze, and predict the chaotic time series in the phase space. The recommendation module is established by using typed parameter analysis and category similarity feature analysis, and the workflow of mobile library resource recommendation based on digital twin technology is shown in Figure 3.

**Figure 4:**
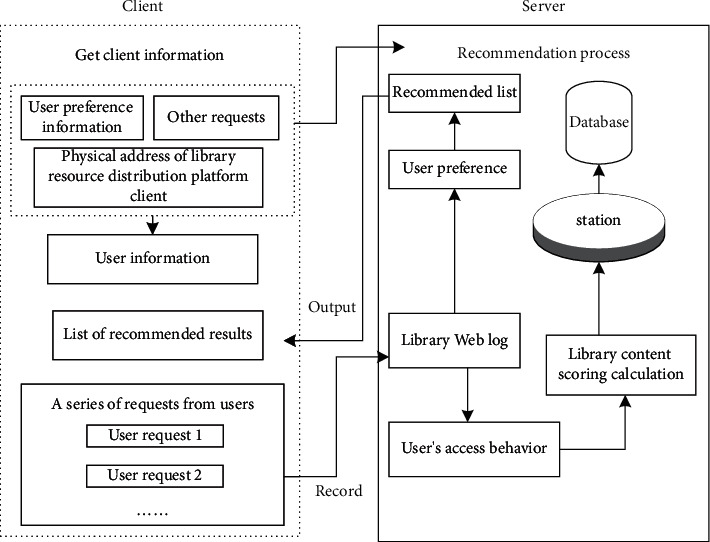
Workflow of resource recommendation of mobile library based on digital twin technology.

According to the distributed structure model of mobile library resource recommendation and sharing resources shown in Figure 3, the phase space reconstruction method is used to reconstruct and extract the features of library resources under the intelligent service mode, and the recommendation module is established by using the typed parameter analysis and category similarity feature analysis [21]. The workflow of mobile library resource recommendation based on digital twin technology is shown in Figure 4.

Establish the resource distribution attribute set *i* ∈ *S*_*s*_ under the intelligent optimization model of mobile library resource recommendation service, and use semantic extraction method to retrieve the information of mobile library resource recommendation sharing. The semantic feature distribution mapping satisfies

(4) $$\begin{array}{c} \alpha^{T}Q\alpha =\sum_{i=1}^{n}\sum_{j=1}^{n}\alpha_{i}\alpha_{j}Q_{ij}\geq 0. \end{array}$$

In formula (4), *α*_*i*_ is the satisfaction parameter of mobile library resource recommendation, *α*_*j*_ is the reliability parameter of mobile library resource recommendation, *Q*_*ij*_ is the personalized feature matching parameter, and the trust relationship of library resources under the intelligent service mode is expressed *A*⟶*B* and *B*⟶*C*. According to the above analysis, the semantic extraction method is used to search the information of mobile library resource recommendation and share, extract the semantic correlation features of mobile library resource recommendation, and filter and recommend the information of mobile library resource recommendation and share.

### 3.2. Semantic Relevance Feature Extraction

**Figure 5:**
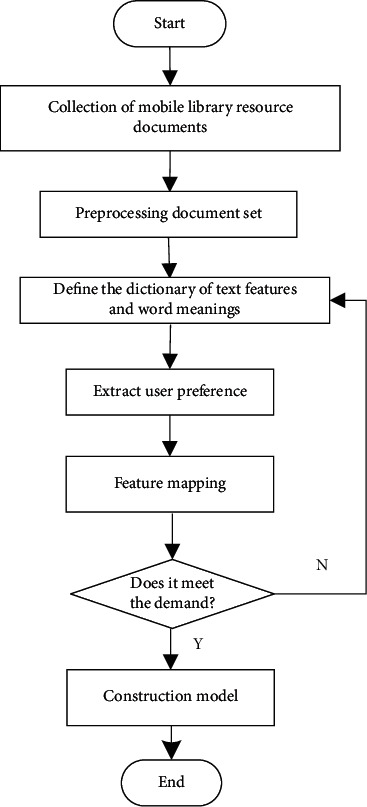
Process of building model.

The semantic extraction method to retrieve information of mobile library resource recommendation and sharing is used, semantic correlation features of mobile library resource recommendation is extracted, a big data statistical analysis model of mobile library resource recommendation and sharing is constructed [22]. The specific process is shown in Figure 5.

Association rules under the intelligent optimization model of mobile library resource recommendation service is scheduled according to the attribute features of library resources, analyzes the distribution feature values of multi-source data recommended by mobile library resources [23], and it makes fuzzy matching according to user preference scores in the recommended cache area, The details are shown in Table 1:

The tag category parameters are established, and the personalized features of mobile library resources are mined by using the feature registration algorithm, and the iterative formula of semantic correlation feature reconstruction of mobile library resources recommendation and sharing is obtained as follows:

(5) $$\begin{array}{c} x_{i}^{\left(k+1\right)}=\left(1-\omega \right)x_{i}^{\left(k\right)}+{\frac{\omega }{a_{n}}}_{i}\left(b_{i}-\overset{i-1}{\underset{j=1}{\sum }}a_{ij}x_{j}^{\left(k+1\right)}-\overset{n}{\underset{j=i+1}{\sum }}a_{ij}x_{j}^{\left(k\right)}\right),\;i=1,2,\ldots ,n,k=1,2,\ldots ,n. \end{array}$$

In formula (5), *ω* is a weighted learning parameter, *x*_*i*_^(*k*)^ and *x*_*j*_^(*k*)^ represent personalized knowledge map and project distribution parameters of mobile library resources, respectively, and *a*_*ij*_ is a fuzzy matching degree. According to the part-of-speech tagging and part-of-speech filtering results of book information, the discrete scheduling feature distribution set of collaborative filtering of library resources is established as follows:

(6) $$\begin{array}{c} S_{b}=\sum_{i=1}^{c}p_{i}\left({\overset{⟶}{m}}_{i}-\overset{⟶}{m}\right){\left({\overset{⟶}{m}}_{i}-\overset{⟶}{m}\right)}^{T}. \end{array}$$

In formula (6), $\overset{⟶}{m}=\sum_{i=1}^{c}p_{i}{\overset{⟶}{m}}_{i}$ is the difference between positive samples and negative samples, *p*_*i*_ is the recommended reliability probability magic parameter, and fuzzy clustering is carried out according to the matching results of semantic features of library resources under the intelligent service mode. The semantic ontology concept set of library resources under the intelligent service mode is as follows:

(7) $$\begin{array}{c} J\left({\overset{⟶}{X}}_{j}\right)=\frac{y_{j}^{T}S_{b}y_{j}}{\lambda_{j}},\;j=1,2,\ldots ,l. \end{array}$$

In formula (7), *y*_*j*_ is the correlation entropy, *λ*_*j*_ is the recommended reliability characteristic value, and *S*_*b*_ is the similarity between recommended combinations. Using autoregressive analysis method to schedule library information under intelligent service mode [24], the descending result of semantic distribution features is obtained: $J\left({\overset{⟶}{X}}_{1}\right)\geq J\left({\overset{⟶}{X}}_{2}\right)\geq \cdots \geq J\left({\overset{⟶}{X}}_{l}\right)$, taking the characteristic quantity with the smallest characteristic value as the fuzzy clustering center of library resources collaborative filtering, the recommended node set of library resources collaborative filtering is $\bar{X_{i}}$, The feature extraction technology is used to extract the average mutual information feature quantity of library resources under the intelligent service mode, and the distance between the centers of two clusters is *y*_*j*_, *y*_*j*_, thus obtaining the semantic relevance feature extraction result of library resources under the intelligent service mode:

(8) $$\begin{array}{c} W=\left[y_{1},y_{2},\ldots ,y_{d}\right]. \end{array}$$

In formula (8), *y*_1_, *y*_2_,…, *y*_*d*_ represents the invariant moment between recommended attribute classes. According to the semantic correlation feature extraction results, collaborative filtering recommendation is carried out under the intelligent optimization model of mobile library resource recommendation service.

## 4. Optimization of Mobile Library Resource Recommendation Algorithm

### 4.1. Mining the Reading User's Object Preference Information and Retrieving the Text Information in the Mobile Library

On the basis of the above-mentioned semantic extraction method for information retrieval of mobile library resource recommendation and sharing, and extracting semantic correlation features of mobile library resource recommendation, the intelligent optimization model of mobile library resource recommendation service is designed. An intelligent optimization model of mobile library resource recommendation service is proposed based on digital twin technology. The semantic fuzziness feature matching method is used to retrieve the text information in the process of resource recommendation and sharing of mobile library [25], and the recommended resources of library are reduced from *M* dimension to *D* dimension in feature space, and the semantic distribution structure model of collaborative recommendation for resource recommendation and sharing of mobile library is obtained:

(9) $$\begin{array}{c} \max F\left(X\right)=\left(F_{1}\left(X\right),F_{2}\left(X\right),\ldots ,F_{n}\left(X\right)\right),\\ \text{s.t}g_{j}\left(X\right)\leq 0\left(j=1,2,\ldots ,p\right)\\ h_{k}\left(X\right)=0\left(k=1,2,\ldots ,p\right). \end{array}$$

In formula (9), *F*_1_(*X*), *F*_2_(*X*),…, *F*_*n*_(*X*) represent the personalized source data of mobile library resources, *g*_*j*_(*X*) is the statistical feature quantity, and *h*_*k*_(*X*) is the feature preference distribution set of recommended items. By adopting differentiated feature preference analysis and combining multi-objective optimization switching, the matching value of the benefit degree of mobile library resource recommendation can be obtained:

(10) $$\begin{array}{c} y_{k}=\sum_{t=0}^{l}w_{lk}x_{lk}. \end{array}$$

In formula (10), *w*_*lk*_ is the benefit distribution parameter of mobile library resource recommendation, *x*_*lk*_ is the adaptive equilibrium coefficient of mobile library resource recommendation, and *l* represents the modeled statistical characteristic quantity. Based on the joint analysis of users, items, and scores, the weighted characteristic value of mobile library resource recommendation is

(11) $$\begin{array}{c} W_{k}={\left[w_{0k},w_{1k},\ldots ,w_{lk}\right]}^{T}. \end{array}$$

In formula (11), *w*_0*k*_ is the weighting coefficient of the *k*th node under the joint constraint of user set and item set, *w*_1*k*_ is the weighting coefficient of the next node, and *w*_*lk*_ is the weighting coefficient of terminal *i* at time *t*.

The mining results of reading user object preference information of mobile library in the process of resource recommendation and sharing of mobile library are as follows:

(12) $$\begin{array}{c} \text{Racall}\left(X,Y\right)=\frac{P\left(X\cap Y\right)}{P\left(X\right)+P\left(Y\right)-P\left(X\cap Y\right)}, \end{array}$$

(13) $$\begin{array}{c} \text{Overload}\left(X,Y\right)=\frac{P\left(X\cap Y\right)}{\text{min}\left(P\left(X\right),P\left(Y\right)\right)}, \end{array}$$

(14) $$\begin{array}{c} \text{Time}\left(X,Y\right)=\frac{2P\left(X\cap Y\right)}{P\left(X\right)+P\left(Y\right)}. \end{array}$$

In formulas (12)–(14), *P*(*X*) and *P*(*Y*) represent the probability density function of the fusion of library information resource recommendation and mobile library reading user object preference under the intelligent service mode, *X* and *Y* are the concept sets of library information resource distribution, and *P*(*X*∩*Y*) is the joint cross distribution set. According to the above analysis, the object preference information mining and text information retrieval of mobile library readers are realized, and the resource recommendation and sharing collaborative filtering recommendation design of mobile library is carried out according to the matching results of the two features.

### 4.2. Mobile Library Resource Recommendation and Sharing Optimization

Establish a data set of association rules for reading users' preferences of mobile library resources recommendation and sharing [26], and use digital twin matching method to block features [27]. The association rules structure model of mobile library resources recommendation and sharing is as follows:

(15) $$\begin{array}{c} X=\left[s_{1},s_{2},\ldots ,s_{K}\right]=\left[\begin{array}{cccc} x_{1} & x_{2} & \cdots & x_{K}\\ x_{1+\tau } & x_{2+\tau } & \cdots & x_{K+\tau }\\ \cdots & \cdot \cdot \cdot & \cdots & \cdots \\ x_{1+\left(m-1\right)\tau } & x_{2+\left(m-1\right)\tau } & \cdot \cdot \cdot & x_{M+\left(m-1\right)\tau } \end{array}\right]. \end{array}$$

In formula (15), *K*=*N* − (*m* − 1)*τ* represents the embedding dimension of mobile library resources search, *τ* is the time delay, *m* is the number of layers of semantic relations of mobile library resources, and *s*_*i*_=(*x*_*i*_, *x*_*i*+*τ*_,…,*x*_*i*+(*m* − 1)*τ*_)^*T*^ is called the semantic ontology feature sequence of mobile library resources. The project scoring item *v* ∈ *N*_*u*_ is introduced. According to the scoring distribution of mobile library resource users' items, the classification distribution law of mobile library resource labels is as follows:

(16) $$\begin{array}{c} {\bar{R}}_{ik}=\sum_{j\in N_{\text{u}}}C_{i,j}^{*}R_{jk}. \end{array}$$

In formula (16), ${\bar{R}}_{ik}$ represents the evaluation value of user *u*_*i*_'s satisfaction with the recommended mobile library resources. The credibility level of *R*_*jk*_ library readers *u*_*j*_ to recommended mobile library resources is indicated. Combined with K-means clustering method, library readers' preference analysis is realized, and the trust value of library resource recommendation under intelligent service mode is obtained:

(17) $$\begin{array}{c} {\text{ITrust}}_{a⟶c}=\frac{\sum_{b\in \text{adj}\left(a,c\right)}{\text{DTrust}}_{a⟶c}\times \left({\text{DTrust}}_{a⟶c}\times \beta_{d}\right)}{\sum_{b\in adj\left(a,c\right)}{\text{DTrust}}_{a⟶b}}. \end{array}$$

In formula (17), Trust_*a*⟶*b*_ represents the trust degree of recommended choices *A* to *B*, and has a similar concept. Trust_*b*⟶*c*_ is the domain ontology parameter. According to the scoring results of *N* mobile library resource users, the typed list parameter analysis is adopted. The digital twin matching method is used to block the features, and the K-means clustering method is used to realize the library reader preference analysis, so as to realize the collaborative matching of mobile library resource recommendation sharing and reading user object preference, and complete the collaborative filtering recommendation of mobile library resource recommendation sharing. The iterative formula of collaborative filtering recommendation of library resources under intelligent service mode is obtained as follows:

(18) $$\begin{array}{c} x_{i}\left(k+1\right)=x_{i}\left(k\right)+s\left(\frac{x_{j}\left(k\right)-x_{i}\left(k\right)}{\left\|x_{j}\left(k\right)-x_{i}\left(k\right)\right\|}\right). \end{array}$$

**Figure 6:**
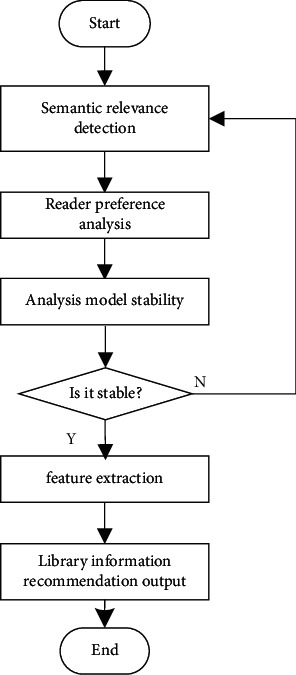
Implementation process of the algorithm.

In formula (18), $\left\|\overset{⟶}{x}\right\|$ represents the norm of $\overset{⟶}{x}$, the preference feature of reading user objects in mobile library is *P*_*ij*_^best^(*k*), and the iteration step is *S*. To sum up, the collaborative filtering recommendation of resource recommendation and sharing of mobile library is realized, and the intelligent optimization model of resource recommendation service of mobile library is optimized according to the optimization design of fusion scheduling algorithm. The implementation process is shown in Figure 6.

## 5. Simulation and Result Analysis

In order to test the application performance of this method in collaborative filtering recommendation of mobile library resource recommendation sharing, an experimental analysis was carried out, and a simulation test was carried out by combining Matlab and C++ programming software. The sample size of information sampling of library resources in intelligent service mode is 2000, the test set size of reading user's preference information distribution in mobile library is 400, and the initial statistic value of intelligent optimization model of mobile library resource recommendation service is ${\hat{\delta }}_{0}=-15$. The number of users is set to 20,000, and 1,500 users are randomly generated as the test set and 200 users as the training set. The recommended parameters *λ*_1_=1, *λ*_2_=1, *c*_1_=2, *c*_2_=2 are adaptively and collaboratively filtered, and the distribution of fuzzy decision parameters for resource recommendation and sharing of mobile library is shown in Table 2. See Table 3 for user parameter configuration.

**Figure 7:**
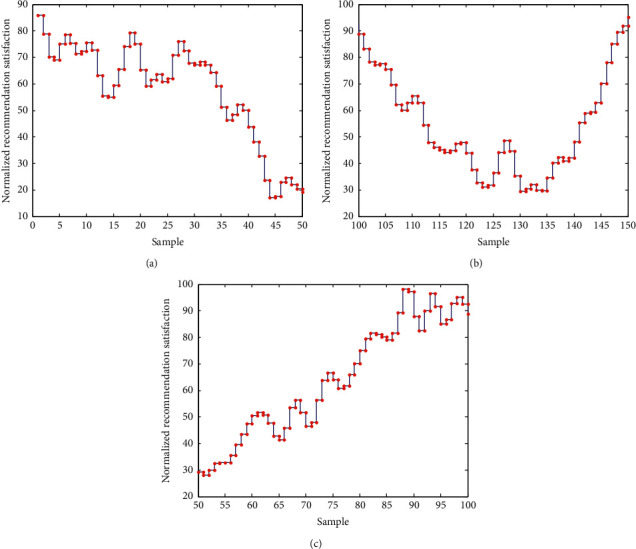
Satisfaction distribution of resource recommendation of mobile library. (a) Evenly distribution (b) Differential distribution. (c) Optimal distribution.

According to the above parameter design results, the collaborative filtering recommendation of mobile library resource recommendation sharing is carried out, and the semantic relevance feature registration is carried out according to the retrieval preference of reading user objects in mobile library, and the user object preference association rule data set pushed by mobile library resources is established. The confidence level distribution weight is set to 1.25, and the mean square error is set to 0.031. The satisfaction level distribution of the recommendation is shown in Figure 7.

According to the analysis of Figure 7, the satisfaction level of mobile library resource recommendation by this method is high, among which the average satisfaction level of recommendation in the optimal state is 95.1%, and the recommendation satisfaction level is higher in the confidence range of *P* < 0.01. The reason is that this method extracts semantic correlation features, which is conducive to improving the satisfaction of mobile library resource recommendation.

**Figure 8:**
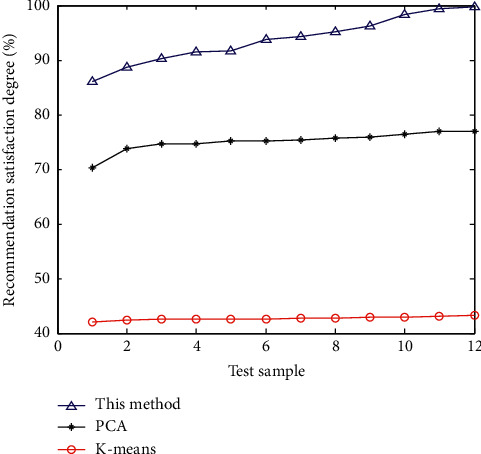
Comparative test of resource satisfaction of mobile library.

The resource holding level of mobile library resources recommended by different methods is tested, and the comparison results are shown in Figure 8.

After analyzing Figure 8, it is found that the adaptability and preference satisfaction level of mobile library resources recommended by this method are high, which improves the user's satisfaction level with mobile library resources. Because of the small number of iteration steps, it can be seen that this method has a good optimization management ability. The reason is that this method is conducive to improving the adaptability and preference satisfaction of mobile library reading user object preference information mining and text information retrieval in the research process.

To sum up, using the intelligent optimization method of Mobile Library Resource Recommendation Service Based on digital twin technology to recommend mobile library resources has a high level of satisfaction, and the average satisfaction of recommendation in the optimal state is 95.1%; It can improve users' satisfaction with mobile library resources and has better optimization management ability.

## 6. Conclusion and Prospect

### 6.1. Conclusion

This paper proposes an intelligent optimization model of Mobile Library Resource Recommendation Service Based on digital twin technology. To realize the collaborative matching between the recommendation and sharing of mobile library resources and the preferences of reading users, the following conclusions are obtained through the research:

1. The method in this paper has good registration for Mobile Library Resource Recommendation and sharing
2. Using this method to recommend mobile library resources has a high level of satisfaction
3. Using this method to recommend mobile library resources has a high level of adaptability and preference satisfaction

### 6.2. Prospect

Further research is still needed for the next step:

1. Each mobile library has its own characteristics, and does not fully use all the models and mechanisms of library resource aggregation and services. Only by properly improving and perfecting the methods and technologies of resource aggregation in combination with the characteristics, advantages and disadvantages of specific libraries, can we realize the deep aggregation and innovative services of mobile library resources on the basis of reflecting the characteristics of the library.
2. The lack of systematic understanding of mobile library resource aggregation and innovative services, the combination of resource aggregation and innovative services, enhances the integrity and systematicness of the research itself in the process of research from a certain angle, thus strengthening the service effect of resource aggregation and innovative services on user resource retrieval and knowledge acquisition, which needs to be further studied in the future.

## Figures and Tables

**Table 1:** Resource recommendation label attribute allocation matrix of mobile library.

Item	Attribute 1	Attribute 2	……	Attribute *j*	……	Attribute *m*
Item 1	LI_11_	LI_12_	……	LI_1*j*_	……	LI_1*m*_
Item 2	LI_21_	LI_22_	……	LI_2*j*_	……	LI_2*m*_
……	……	……	……	……	……	……
Item *i*	LI_*i*1_	LI_*i*2_	……	LI_*ij*_	……	LI_*im*_
……	……	……	……	……	……	……
Item *n*	LI_*n*1_	LI_*n*2_	……	LI_*nj*_	……	LI_*nm*_

**Table 2:** Fuzzy decision parameters of resource recommendation and sharing in mobile library.

*X* Value	*Y* Value	Fuzzy closeness vector *Q*_1_, *Q*_2_, *Q*_3_, *Q*_4_, *Q*_5_	Priority order of recommendation and sharing of mobile library resources
821.404	417.343	0.334	*Q*_4_ > *Q*_1_ > *Q*_3_ > *Q*_2_ > *Q*_5_
262.254	359.380	0.504	*Q*_3_ > *Q*_2_ > *Q*_1_
89.749	887.285	0.480	*Q*_4_ > *Q*_1_ > *Q*_3_ > *Q*_2_ > *Q*_5_
479.380	396.897	0.118	*Q*_4_ > *Q*_1_ > *Q*_3_ > *Q*_2_ > *Q*_5_
149.439	381.151	0.328	*Q*_4_ > *Q*_1_ > *Q*_3_ > *Q*_2_ > *Q*_5_
233.175	707.076	0.869	*Q*_4_ > *Q*_1_ > *Q*_3_ > *Q*_2_ > *Q*_5_
519.723	150.878	0.810	*Q*_4_ > *Q*_1_ > *Q*_3_ > *Q*_2_ > *Q*_5_

**Table 3:** User parameter configuration.

User list	Preference level	Contribution degree	Similarity level
1	0.166	0.166	5.3428
2	0.167	0.204	7.4065
3	0.169	0.146	4.8596
4	0.177	0.161	1.0726
5	0.171	0.187	1.5970
6	0.176	0.174	7.9761
7	0.171	0.187	1.6630
8	0.176	0.177	6.2025
9	0.173	0.207	6.3593
10	0.174	0.186	3.0750

## Data Availability

The raw data supporting the conclusions of this article will be made available by the authors, without undue reservation.
